# Role of Histidine 547 of Human Dopamine Transporter in Molecular Interaction with HIV-1 Tat and Dopamine Uptake

**DOI:** 10.1038/srep27314

**Published:** 2016-06-02

**Authors:** Yaxia Yuan, Pamela M. Quizon, Wei-Lun Sun, Jianzhuang Yao, Jun Zhu, Chang-Guo Zhan

**Affiliations:** 1 Molecular Modeling and Biopharmaceutical Center and Department of Pharmaceutical Sciences, College of Pharmacy, University of Kentucky, 789 South Limestone Street, Lexington, KY 40536, USA; 2 Department of Drug Discovery and Biomedical Sciences, South Carolina College of Pharmacy, University of South Carolina, 715 Sumter Street, Columbia, SC 29208, USA.

## Abstract

HIV-1 Tat plays an important role in HIV-associated neurocognitive disorders (HAND) by disrupting neurotransmission including dopamine uptake by human dopamine transporter (hDAT). Previous studies have demonstrated that HIV-1 Tat directly binds to hDAT and some amino-acid mutations that attenuate the hDAT-Tat binding also significantly decreased dopamine uptake activity of hDAT. This combined computational-experimental study demonstrates that histidine-547 (H547) of hDAT plays a crucial role in the hDAT-Tat binding and dopamine uptake by hDAT, and that the H547A mutation can not only considerably attenuate Tat-induced inhibition of dopamine uptake, but also significantly increase the V_max_ of hDAT for dopamine uptake. The finding of such an unusual hDAT mutant capable of both increasing the V_max_ of hDAT for dopamine uptake and disrupting the hDAT-Tat binding may provide an exciting knowledge basis for development of novel concepts for therapeutic treatment of the HAND.

According to the 2013 report of World Health Organization (WHO), about 35.3 million people in the world live with the acquired immune deficiency syndrome (AIDS) disease caused by human immunodeficiency virus (HIV)^1^, and about 70% of HIV-infected individuals suffer from HIV-associated neurocognitive disorders (HAND)^2^,^3^,^4^,^5^. Within the genes of HIV virus, the transactivator of transcription (Tat) gene plays a crucial role in regulation of proteins that control how the HIV virus infects cells^2^,^6^,^7^,^8^. It has been known that HIV-1 Tat, detected in the brain and the sera of HIV-1 patients^9^,^10^,^11^, plays an important role in HAND by disrupting neurotransmission^12^ including dopamine uptake by human dopamine transporter (hDAT). Presynaptic hDAT activity is strikingly reduced in HIV-1 patients, particularly those with cocaine abuse^13^,^14^. Recently reported computational and experimental studies^15^,^16^,^17^ examined how HIV-1 Tat interacts with hDAT at molecule level, demonstrating that HIV-1 Tat directly binds to hDAT and that amino-acid residues Y88, K92, and Y470 of hDAT are involved in the hDAT-Tat binding. The K92M, Y470H, and Y470A mutations all significantly attenuated Tat-induced inhibition of dopamine uptake. Meanwhile, these mutations also decreased the V_max_ of hDAT for dopamine uptake^15^,^16^,^17^. Here we demonstrate that H547 is also involved in the hDAT-Tat binding, and that the H547A mutation can not only considerably attenuate Tat-induced inhibition of dopamine uptake, but also significantly increase the V_max_ of hDAT for dopamine uptake. The unusual H547A mutation on hDAT was proposed based on computational modeling of the detailed three-dimensional (3D) structures, followed by *in vitro* pharmacological testing. The finding of such an unusual hDAT mutant capable of increasing the V_max_ of hDAT for dopamine uptake while effectively attenuating Tat-induced inhibition of dopamine uptake may provide an exciting knowledge basis for development of novel concepts for therapeutic treatment of the HAND.

## Results

### Role of H547 in hDAT binding with Tat

**Figure 1:**
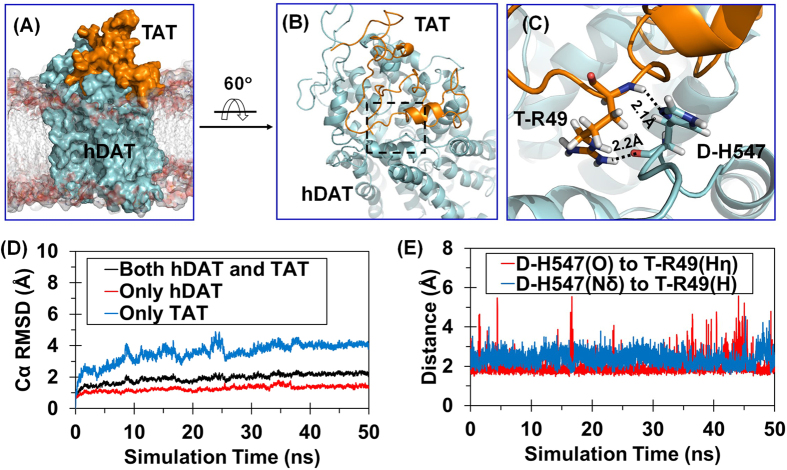
MD-simulated hDAT-Tat binding structure. (**A**) Overview of MD-simulated outward-open hDAT-Tat complex structure in the lipid bilayer. The hDAT and Tat structures are represented as molecular surface in cyan and orange, respectively. The side chains of lipid molecules are shown in semi-transparent stick-and-ball style, and the head group atoms as semi-transparent molecular surface. (**B**) hDAT and Tat are represented as cartoon in cyan and orange, respectively. Details of local structure in the dashed box are showed in panel C. The lipid molecules are wiped off for clarity. (**C**) Local view of the extracellular loop 6 of hDAT and Tat. Dashed lines indicate the hydrogen bonds between T-R49 and D-H547 with key distances labeled; the prefix D- and T- indicate hDAT and Tat, respectively. (**D**) Tracked changes for the positional root-mean-square deviations (RMSD) for the Cα atoms of the outward-open hDAT-Tat complex structure (black curve), the hDAT component (red curve), and the Tat component (blue curve) along the MD simulation. (**E**) Tracked distances involved in the hydrogen bonds between D-H547 and T-R49.

As well known, hDAT may exist in three typical conformational states in the dopamine-transporting cycle: the outward-open state (*i.e.* the extracellular side of binding site for the transmitter is open, while the intracellular side is blocked), the outward-occluded state (*i.e.* both the extracellular and intracellular sides of binding site are blocked such that the binding site is occluded and no longer accessible for substrate), and the inward-open state (*i.e.* the intracellular side of binding site for the transmitter is open, while the extracellular side is blocked)^18^,^19^,^20^,^21^,^22^,^23^,^24^. As demonstrated in our previous studies^15^, Tat binds most favorably with the outward-open state of hDAT and, thus, blocks dopamine uptake by preventing the conformational conversion of hDAT from the outward-open state (Fig. 1A) to the other states during the dopamine-transporting cycle^25^. According to further molecular dynamics (MD) simulation (50 ns) on the outward-open hDAT-Tat complex in the present study, another residue, *i.e.* histidine 547 (H547), of hDAT plays a crucial role in its binding with Tat. H547 exists in the extracellular loop 6 (EL6) which is important for the stability of hDAT-Tat binding complex (Fig. 1B,C).

Depicted in Fig. 1D is the tracked change of positional root-mean-square deviation (RMSD) for the Cα atoms of the outward-open hDAT-Tat complex (black curve) from the starting structure used for the MD relaxation, along with the calculated RMSD values for the hDAT component (red curve) and Tat component (blue curve) in the complex. As seen in Fig. 1D, after ~25 ns, all of the RMSD curves became flat, indicating that the MD-simulated hDAT-Tat complex structure was equilibrated very well after ~25 ns. Although Tat had much larger RMSD values than hDAT due to the higher flexibility of Tat structure, two hydrogen bonds between H547 of hDAT (denoted as D-H547 for convenience, with the prefix D- indicating hDAT) and R49 of Tat (denoted as T-R49 for convenience, with the prefix T- indicating Tat) were stable during the entire MD simulation process. Depicted in Fig. 1E are the tracked internuclear distances between the Nδ atom of D-H547 side chain and hydrogen atom of T-R49 backbone and between the carbonyl oxygen atom of D-H547 backbone and the Hη atom of T-R49 side chain. So, H547 is crucial for hDAT binding with Tat. Mutation of H547 to another residue, such as A, is expected to disrupt the hydrogen bond with D-H547 side chain and, thus, considerably weaken the hDAT-Tat binding.

### A structural motif (Y548-Y470-Y551) crucial for dopamine transporting in hDAT

**Figure 2:**
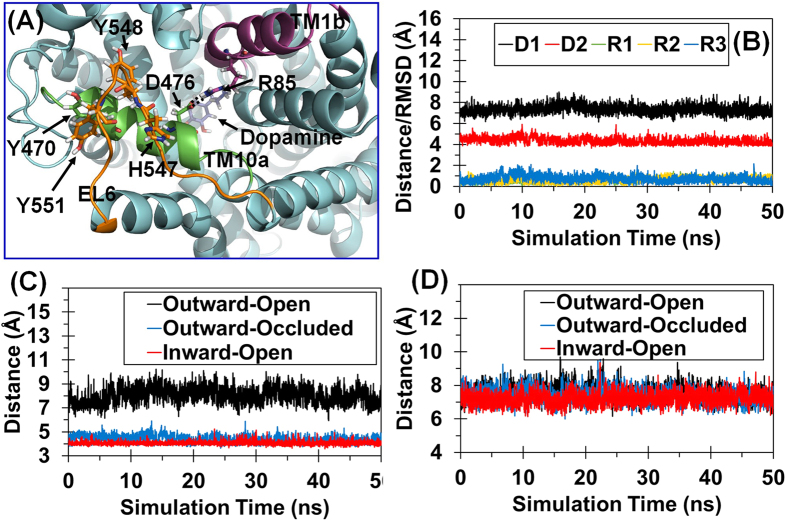
MD-simulated WT hDAT structures in the three typical conformational states. (**A**) Structural model of WT hDAT in the outward-occluded state. hDAT is in cyan cartoon style. TM1b (the second part of transmembrane helix 1), TM10a, and EL6 are in purple, green, and orange, respectively. Dopamine and key residues are showed in stick-ball style. Hydrogen bonds between D476 and R85 are indicated in dashed lines. (**B**) Tracked distances and positional RMSD values for WT hDAT. D1 indicates the tracked internuclear distance between the Cα atoms of Y470 and Y548. D2 refers to the tracked internuclear distance between Cγ atom of D476 and the Cζ atom of R85. The tracked changes of the RMSD for the Cα atoms of Y470, R85, and D476 are indicated by R1, R2, and R3, respectively. (**C**) Tracked internuclear distance between the Cγ atom of D476 and the Cζ atom of R85 in three typical conformational states of WT hDAT. (**D**) Tracked internuclear distance between the Cα atoms of Y470 and Y548 in three typical conformational states of WT hDAT.

Previous studies^26^,^27^,^28^,^29^ revealed an essential structural feature during the conformational conversion of hDAT from the outward-open state to the other states during the dopamine transporting process, *i.e.* formation of a stable D476-R85 salt bridge (Fig. 2A) in hDAT. The D476-R85 salt bridge exists in the outward-occluded and inward-open states, but not in the outward-open state. So, formation of the D476-R85 salt bridge is the key to the conformational conversion of hDAT from the outward-open state to the outward-occluded state. This essential feature is well-reflected in our MD simulations on hDAT in three typical conformational states. For example, based on the MD simulations, D476 (in the first part of transmembrane helix 10, denoted as TM10a) was far away from R85 (in the second part of transmembrane helix 1, denoted as TM1b) in the outward-open state with an average distance of 7.76 ± 0.57 Å (black curve in Fig. 2C) between the Cγ atom of D476 and the Cζ atom of R85, indicating no direct interaction between these two residues in the outward-open state. In comparison, the tracked distance between the Cγ atom of D476 and the Cζ atom of R85 became as short as 4.30 ± 0.31 Å (blue curve in Fig. 2C) in the outward-occluded hDAT-DA binding structure and even shorter (4.05 ± 0.14 Å, red curve in Fig. 2C) in the inward-open hDAT-DA binding structure.

**Figure 3:**
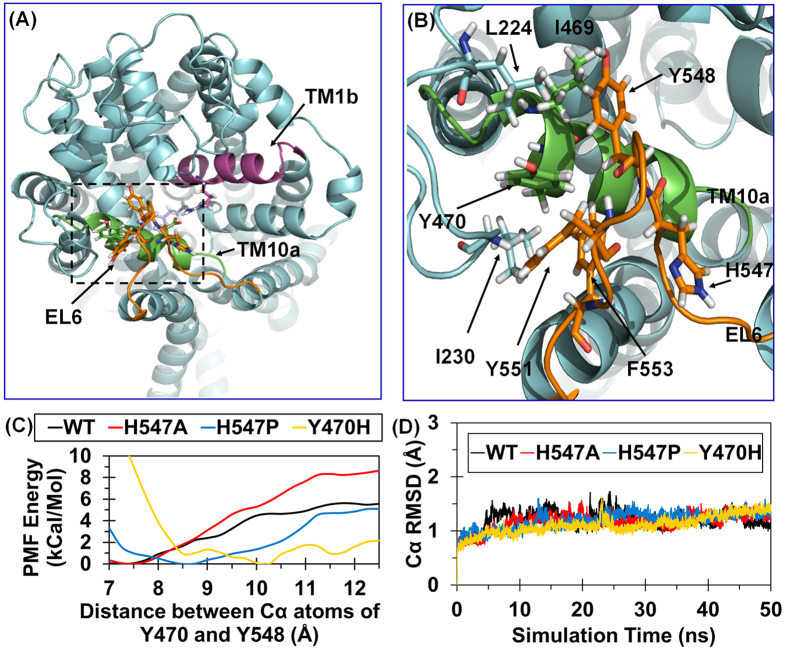
The Y548-Y470-Y551 motif identified from computational modeling. (**A**) Overview model of WT hDAT in the outward-occluded state. hDAT is in cyan cartoon style. TM1b, TM10a, and EL6 are in purple, green, and orange, respectively. Details of local structure in the dashed box is showed in panel B. (**B**) Local view of the hydrophobic region around Y470. Key hydrophobic residues and H547 are showed in stick-ball style. (**C**) PMF-simulated free energy profile of Y548 unbinding from Y470 as a function of distance between the Cα atoms of Y548 and Y470. (**D**) Tracked changes of the positional RMSD values for the Cα atoms of WT hDAT and its H547A, H547P, and Y470H mutants in the outward-occluded state.

In further analysis of the MD trajectories for all of the three typical conformational states of hDAT (wild-type and some mutants to be discussed below), the conformational conversion of hDAT from the outward-open state to the outward-occluded state and then to the inward-open state requires existence of a stable structural motif (*i.e*. Y548-Y470-Y551, denoted as YYY motif). Specifically, according to our previously reported homology model^25^ of hDAT based on drosophila DAT (PDB entry code: 4M48, resolution: 2.95 Å)^30^, a U-turn loop (residues #547 to #552: HYGAYI) was observed, as noted in EL6 (Fig. 3A), and this U-turn loop has hydrophobic contact with Y470. In this local hydrophobic region including L224, I230, I469, Y470, Y548, Y551, and F553 (Fig. 3B), the aromatic side chains of residues Y548 and Y551 (all in EL6) pack the aromatic side chain of Y470 (in TM10a) in a way like a plier, which is expected to stabilize Y470 side chain and, thus, anchor TM10a. Notably, such a stable YYY motif exists in all of the three typical conformational states of hDAT during the dopamine transporting process. As discussed below, certain amino-acid mutations that destabilize the YYY motif also impair the formation of the D476-R85 salt bridge and, thus, make the conformational conversion associated with the dopamine transporting process more difficult. So, the YYY motif plays an essential role in the required conformational conversion of hDAT during dopamine transporting process.

Based on the structural feature of the YYY motif, mutating Y470 to a hydrophilic amino acid with a similar size and shape, such as the Y470H mutation, would destabilize the YYY motif due to the disruption of the hydrophobic interaction, which is supported by our previously reported fact that the Y470H mutation considerably decreased the V_max_ of hDAT for dopamine uptake^16^,^17^. In addition, because H547 is involved in the U-turn loop and adjacent to Y548 of the YYY motif, a single-point mutation on H547 (such as H547A or H547P mutation) that can alter the backbone conformational tendency is also expected to alter the conformation of the U-turn loop and, thus, influence the strength of the YYY motif.

### Effects of amino-acid mutations on the YYY motif and D476-R85 salt bridge

The strength of the YYY motif is determined by both the U-turn loop conformation (which is the critical environment of the YYY motif) and the hydrophobic interactions among the aromatic side chains of the three tyrosine residues within the YYY motif itself. Hence, a mutation on the first residue (H547) of the U-turn loop or Y470 within the YYY motif is expected to significantly affect the stability of the YYY motif. In order to computationally examine possible effects of certain mutations on the YYY motif stability, we estimated the free energy (denoted as unbinding free energy) required to separate Y548 from Y470 by using potential of mean force (PMF) approach^31^,^32^. The PMF approach was used to determine the free energy profile of the unbinding process (*i.e*. the separation of Y548 from Y470). For calculation of the unbinding free energy using the PMF approach, it is necessary to define a “reaction coordinate” which is the internuclear distance between the Cα atoms of H470 and Y548. The longest possible Cα-Cα distance between Y548 and Y470 was founded to be 10.25 Å in the MD-simulated Y470H mutant structure. So, the Cα-Cα distance of 10.25 Å was used as the unbinding state in our unbinding free energy calculations.

Depicted in Fig. 3C are the unbinding free energy profiles obtained for wild-type (WT) hDAT along with the H547A, H547P, and Y470H mutants. As seen in Fig. 3C, in the Y470H mutant, the calculated unbinding free energy should be zero (0 kcal/mol) because the equilibrium Cα-Cα distance in the Y470H mutant is defined as that in the unbinding state. The relative unbinding free energies for the WT, H547A, H547P, and Y470H mutants were estimated to 4.65, 5.78, 1.70, and 0 kcal/mol, respectively (Table 1). Based on the estimated unbinding free energies, both the H547P and Y470H mutations significantly destabilize the YYY motif, whereas the H547A mutation helps to stabilize the YYY motif, compared to the WT hDAT.

**Figure 4:**
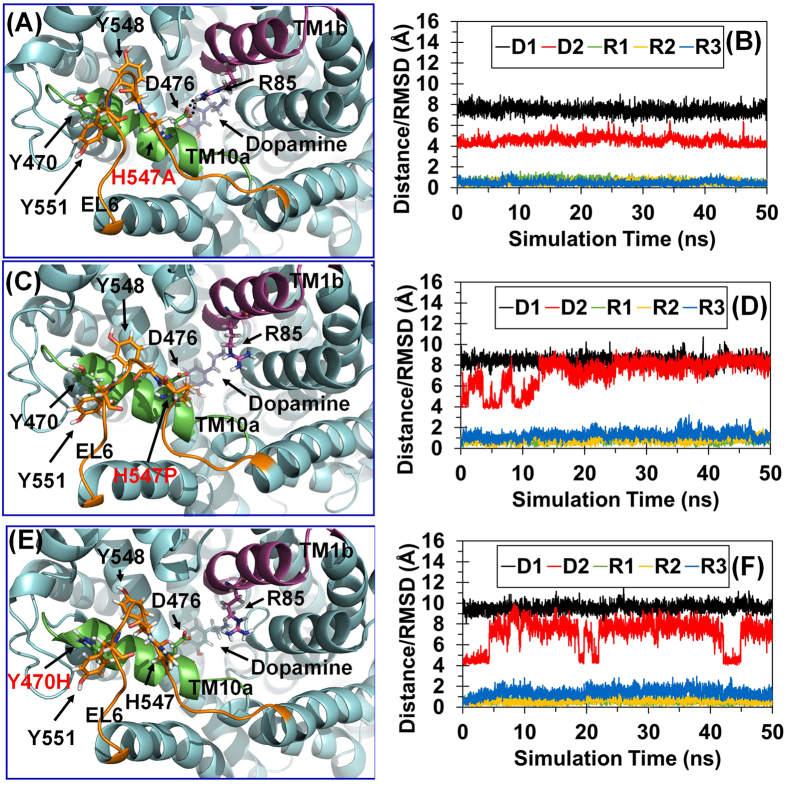
MD-simulated structures of the H547A, H547P, and Y470H mutants of hDAT in the outward-occluded state. (**A**) Structural model of the H547A mutant in the outward-occluded state. hDAT is in cyan cartoon style. TM1b, TM10a, and EL6 are in purple, green, and orange, respectively. Key residues and dopamine are showed in stick-ball style. Hydrogen bonds between D476 and R85 are showed in dashed lines. (**B**) Tracked distances and positional RMSD values for the H547A mutant. D1 indicates the tracked internuclear distance between the Cα atoms of Y470 and Y548. D2 refers to the tracked internuclear distance between the Cγ atom of D476 and the Cζ atom of R85. The tracked changes of the RMSD for the Cα atoms of Y470, R85, and D4716 are indicated by R1, R2, and R3, respectively. (**C**) Structural model of the H547P mutant in the outward-occluded state. (**D**) Tracked distances and positional RMSD for the H547P mutant. (**E**) Structural model of the Y470H mutant in the outward-occluded state. (**F**) Tracked distances and positional RMSD for the Y470H mutant.

To further understand the differential effects of the H547A, H547P, and Y470H mutations on the YYY motif stability in structural detail, we further analyzed the detailed geometric parameters in the MD-simulated structures of the WT hDAT and its H547A, H547P, and Y470H mutants in the outward-occluded state. Figure 3D depicts the tracked changes of positional RMSD for the Cα atoms of the four protein systems. After a time period of ~20 ns, all of the RMSD curves became flat, indicating that these systems were equilibrated very well after ~20 ns. The equilibrated distances between the Cα atoms of Y470 and Y548 in the outward-open, outward-occluded, and inward-open states of WT hDAT were 7.37 ± 0.36 Å, 7.23 ± 0.35 Å, and 7.29 ± 0.37 Å, respectively, suggesting that the YYY motif does not change during the conformational conversion of WT hDAT (Fig. 2D). As a result, the YYY motif may be considered as a static structural feature of WT hDAT. However, the MD simulations revealed that the H547P and Y470H mutations would induce significant conformational change to the YYY motif of hDAT in the outward-occluded state (Fig. 4C–F). The average distances between the Cα atoms of Y470 and Y548 in the WT hDAT, H547A, H547P, and Y470H were 7.37 ± 0.40 Å, 7.26 ± 0.39 Å, 8.40 ± 0.50 Å, and 9.57 ± 0.35 Å, respectively (Table 1), implying that the YYY motif was instable in the H547P and Y470H mutants. All of the computational data consistently suggest that the H547P and Y470H mutations would destabilize the natural YYY motif in hDAT.

As mentioned above, formation of the D476-R85 salt bridge is an essential structural feature for the outward-occluded and inward-open states of WT hDAT. It is interesting to note that the D476-R85 salt bridge was not stable in the MD-simulated structures of the H547P and Y470H mutants in the outward-occluded state, and that the salt bridge was stable in the MD-simulated structures of the WT hDAT and the Y547A mutant (see Figs 2 and 4). The average distances between the Cγ atom of D476 and the Cζ atom of R85 in the WT hDAT, H547A, H547P, and Y470H structures were 4.35 ± 0.36 Å, 4.25 ± 0.25 Å, 8.31 ± 0.42 Å, and 7.61 ± 0.62 Å, respectively (Table 1). These data imply that the D476-R85 salt bridge in the H547A mutant was comparable to that in WT hDAT, whereas the salt bridge was broken in the H547P and Y470H mutants. So, the H547P and Y470H mutations indirectly disrupt the essential D476-R85 salt bridge in the outward-occluded state of hDAT and, thus, impair dopamine uptake by hDAT.

Based on the above observations, the strength of the YYY motif affects the stability of the D476-R85 salt bridge. The tracked RMSD values (~0.53–0.77 Å) for the positions of the Cα atoms of R85 and Y470 (Table 1) in WT hDAT and the three mutants did not significantly change during the MD simulations, whereas the RMSD values (~1.17–1.27 Å) for the positions of the Cα atom of D476 in the H547P and Y470H mutants were significantly larger, indicating that disruption of the D467-R85 salt bridge in the H547P and Y470H mutants in the outward-occluded state is mainly attributed to the unnatural shifting of D476 (in TM10a). Therefore, maintaining the stability of D476 and TM10a is essential for the dopamine uptake activity of hDAT. Furthermore, as the YYY motif plays a key role in anchoring TM10a, there is a positive correlation between the strength of the YYY motif and the stability of TM10a and D476. Due to the relatively lower strength of the YYY motif in the H547P and Y470H mutants, the H547P and Y470H mutations would decrease the dopamine transporting efficiency of hDAT. On the contrary, considering that the YYY motif is an essential static structural feature of hDAT, the relatively more stable YYY motif in the H547A mutant is expected to improve the dopamine-transporting function.

### Experimental validation

Computational results discussed above suggest that H547 of hDAT plays a crucial role in hDAT binding with HIV-1 Tat and dopamine uptake by hDAT, and that the H547P and Y470H mutations are expected to decrease the dopamine uptake activity of hDAT, whereas the H547A mutation is expected to improve the dopamine uptake activity of hDAT. In order to validate the computational predictions, we carried out wet experimental studies including site-directed mutagenesis and dopamine (DA)-uptake assay (see the Methods section for the experimental details) to determine the inhibitory effects of Tat on the dopamine uptake by the H547A mutant in comparison with that by WT-hDAT under the same experimental conditions. In addition, the same type of control experiments described earlier^16^ were carried out, showing no significant difference in the surface expression of hDAT between the wild-type and mutants in cultured cells.

**Figure 5:**
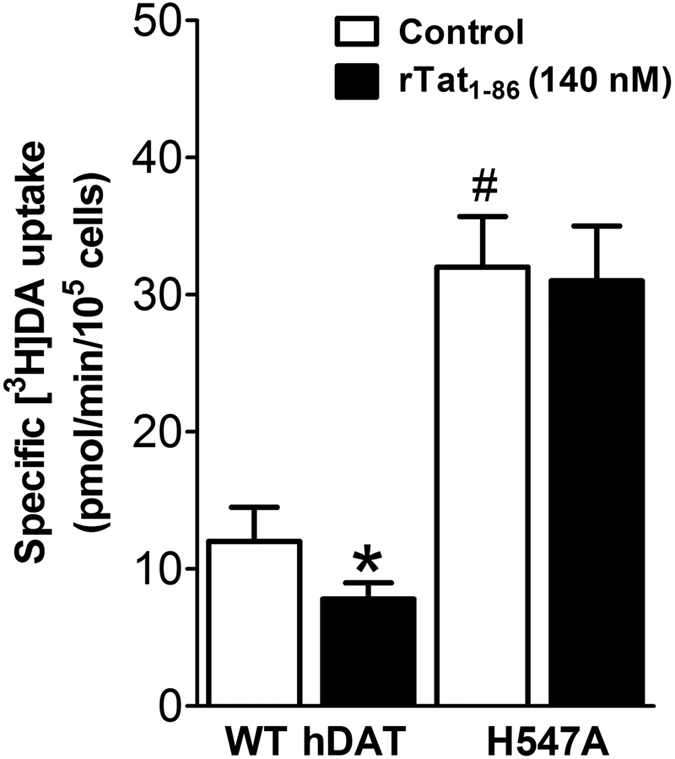
Kinetic analysis on WT hDAT and its mutants in the presence or absence of Tat. Effects of Tat on [^3^H]DA uptake activity of WT hDAT and the H547A mutant. PC12 cells transfected with the WT hDAT or the H547A-hDAT were preincubated with or without recombinant Tat_1–86_ (rTat_1–86_, 140 nM, final concentration) at room temperature for 20 min followed by the addition of a varying concentration of [^3^H]DA (one of six different concentrations). In parallel, nonspecific uptake at each concentration of [^3^H]DA (in the presence of 10 μM nomifensine, final concentration) was subtracted from total uptake to calculate DAT-mediated uptake. Data are expressed as means from four independent experiments ± S.E.M. *p < 0.05 compared to control values (in the absence of Tat). #*p* < 0.05 compared to WT hDAT (n = 4). *p < 0.05 compared to WT hDAT (unpaired Student’s *t* test).

The data depicted in Fig. 5 revealed that, compared to the respective controls, exposure to 140 nM Tat_1–86_ significantly decreased the DA uptake by WT hDAT by 35%, whereas there was no significant effect of 140 nM Tat_1–86_ on the DA uptake by H547A-hDAT, demonstrating that the H547A mutation indeed considerably attenuated Tat-induced inhibition of DA uptake. Further experimental validation was carried out to determine the effects of the H547A, H547P, and Y470H mutations on the dopamine uptake activity of hDAT under the same experimental conditions. As depicted in Table 2, the H547P mutation considerably decreased the V_max_ of hDAT for DA uptake by ~98% (from ~12.4 pmol/min/10^5^ cells to ~0.2 pmol/min/10^5^ cells). The Y470H mutation also had a similar effect on the V_max_ of hDAT for DA uptake^16^, and decreased the V_max_ of hDAT for DA uptake by ~82% (from ~12.4 pmol/min/10^5^ cells to ~2.2 pmol/min/10^5^ cells), as seen in Table 2. In contrast, the H547A mutation significantly increased the V_max_ of hDAT for DA uptake by ~3-fold, compared to that by WT hDAT. The experimental activity data strongly support the computationally predicted role of H547 in hDAT binding with HIV-1 Tat and in dopamine uptake by hDAT.

## Discussion

All of the computational and experimental data presented above consistently demonstrate that H547 of hDAT plays a crucial role of in hDAT binding with HIV-1 Tat and in dopamine uptake by hDAT, and that the H547A mutation can not only significantly attenuate Tat-induced inhibition of DA uptake by disrupting the hDAT-Tat binding, but also significantly improve the dopamine uptake activity of hDAT. The observed attenuation of the Tat-induced inhibition of DA uptake by the H547A mutation also suggests that the H547A mutation can effectively disrupt the hDAT-Tat binding, although a further co-immunoprecipitation (co-IP) experiment is needed to verify/confirm the disruption of the hDAT-Tat binding. In any event, the consistency between the computational and experimental data suggests that the computational insights into the molecular mechanisms of the hDAT-Tat interactions and dopamine uptake by hDAT are reasonable.

Based on the computational and experimental observations of the improved dopamine uptake activity of the H547A mutant, one can realistically expect to further simultaneously improve the dopamine uptake activity of hDAT and disrupt the unwanted hDAT-Tat binding through rational protein engineering design based on the detailed mechanistic understanding. Further, in terms of the rational protein engineering design strategy, it is reasonable to assume that the conformational conversion pathway of an important neurotransmitter transporter, such as hDAT, is highly optimized *via* natural evolution. This means that the amino-acid residues selected naturally to directly interact with dopamine in each step of the transporting cycle are nearly optimal. Therefore, any mutations on any key residue that make the hDAT favor to a certain conformational state would likely decrease the dopamine uptake activity of hDAT, which is supported by many hDAT mutation experiments^16^,^25^,^33^,^34^,^35^. From this perspective, it might be a more promising strategy for improving dopamine uptake activity of hDAT to enhance the stability of some crucial structural units (such as the YYY motif) that do not significantly change during the transporting cycle.

The unusual outcomes, along with the new structural and mechanistic insights, obtained from this study may help to develop novel concepts for future therapeutic treatment of the HAND. For example, it has been known that the HAND-related abnormal neurocognitive function is associated with dysfunctions in dopamine neurotransmission^36^,^37^,^38^. It might be possible to develop a gene therapy for this patient population through effective delivery of a gene for an engineered hDAT mutant (such as one including the H547A mutation) with a significantly improved dopamine update activity without binding with HIV-1 Tat. For development of an effective gene therapy for the HAND treatment, it is essential to have an hDAT mutant which does not significantly bind with Tat so that Tat will not significantly affect the dopamine uptake activity of the hDAT mutant. In addition, the higher the dopamine uptake activity of the hDAT mutant, the more effective the gene therapy would be. Alternatively, one may develop a possible small-molecule drug which can bind with hDAT to affect the hDAT functions in a way similar to the effects of the H547A mutation and, thus, effectively block the hDAT binding with Tat and enhance/keep the normal dopamine uptake activity of native hDAT.

In addition, we note that the YYY motif observed in hDAT also exists in most members of the human NSS family (Table S1) and in DAT proteins of nearly all species (Table S2). Thus, the YYY motif (or a close variant) may also have a similar role in their natural transporting functions of other transporters, and it is possible that enhancement of the YYY motif (or a close variant) may potentially improve their transporting activities of the other members of the NSS family, including those that may also be involved in the HAND. Hence, the similar therapeutic concepts mentioned above could also be useful with other transporters.

## Methods

### Molecular dynamics simulation

Based on the constructed complex structure (outward-open hDAT-Tat) in our previous work^25^, further longer MD simulation was performed using the AMBER 12 software package^39^. The starting structure was gradually heated to 300 K by applying Langevin dynamics and equilibrated for 50 ns. Similarly long MD simulations were also carried out on WT hDAT in three typical conformational states, and the H547A, H547P, and Y470H mutants of hDAT in the outward-occluded state.

The initial structures of the H547A, H547P, and Y470H mutants in the outward-occluded state were generated by using the tLeap module of the AMBER 12 package from the equilibrated structure of WT hDAT in the outward-occluded state. Each protein system was subjected to multiple steps of energy minimization, including 100,000 steps of minimization with the steepest descent method and then 100,000 steps of energy minimization with the conjugated gradient method. In order to remove unexpected steric hindrance induced by the computational mutation operation, an additional force with a force constant of 50 kcal/mol was applied as a constraint first to all atoms of protein and lipid bilayer while the atomic positions of remaining solvent molecules were energy-minimized. Here, for convenience, the protein environment includes all of the atoms of lipid molecules, solvent water molecules, and Na^+^ and Cl^−^ ions. The constraint was then applied to all atoms of the protein (except the mutated residue), while the atomic positions of the protein environment were energy-minimized. In the third step, the constraint was applied to the atomic positions of protein backbone, while all of the other atomic positions were energy-minimized. Finally, the whole system was energy-minimized without any constraint.

During the MD simulations, a 10 Å non-bonded interaction cutoff and 2.0 Å non-bonded list updating cutoff were used. The motion for the mass center of the system was removed every 1000 steps. The particle-mesh Ewald (PME) method^40^,^41^ was used to treat long-range electrostatic interactions. The lengths of covalent bonds involving hydrogen atoms were fixed with the SHAKE algorithm^42^, enabling the use of a 2-fs time step to numerically integrate the equations of motion. For MD simulation on this particular membrane system, a constant surface tension (15 dyne/cm) with interfaces in the X-Y plane was applied. The production MD was kept running for about 50 ns with a periodic boundary condition in the NTP (constant temperature and pressure) ensemble at T = 300 K.

### PMF simulation and unbinding free energy calculation

The calculation of the unbinding free energy between Y548 and Y470 was based on the potential of mean force (PMF) simulations along a “reaction” (separation) coordinate between the Cα atoms of Y548 and Y470. The energy-minimized structures of WT hDAT and its H547A, H547P, and Y470H mutants obtained from the MD simulations mentioned above were used as the starting structures of the unbinding free energy calculations. Each protein system was equilibrated for a total of 10 ns in the NPT ensemble (with the constant temperature and constant pressure). Then, the steered molecular dynamics (SMD) module^43^ implemented in the AMBER 12 package was used to simulate the thermodynamic unbinding pathway. A pulling force with a virtual spring constant of 10.0 kcal/mol/Å^2^ was imposed on the Cα atoms of Y548 and Y470, and the motion speed was 0.0005 Å/ps. To obtain more reasonably simulate the unbinding pathway, four independent trajectories were carried out for each protein system, and the trajectory with the lowest work function was selected as that associated with the most reasonable unbinding pathway for further umbrella sampling, as the lower work function in SMD implies the corresponding pulling process is closer to the quasistatic process. The selected unbinding pathway for each system was split into 23 representative structures along the separation coordinate (23 windows, one representative structure for each window). The range of the distance between the Cα atoms of Y548 and Y470 was from 7.0 Å to 12.5 Å, with a distance interval of 0.25 Å between the adjacent representative structures. A biased potential function with harmonic force constant of 20.0 kcal/mol/Å^2^ was imposed on each of the representative structures. For each window, the structure was subjected to a 200 ps equilibration, followed by 2 ns production MD run. Convergence of the simulation was examined by extending the simulation time for each system. The weighted histogram analysis method (WHAM)^44^ was used to combine all windows to compute the PMF.

### Construction of plasmids

All mutants of hDAT were generated from WT hDAT sequence (NCBI, cDNA clone MGC: 164608 IMAGE: 40146999) by site-directed mutagenesis. Synthetic cDNA encoding hDAT subcloned into pcDNA3.1+ (provided by Dr. Haley E Melikian, University of Massachusetts) was used as a template to generate mutants using QuikChange™ site-directed mutagenesis Kit (Agilent Tech, Santa Clara CA). The sequence of the mutant construct was confirmed by DNA sequencing at the University of South Carolina EnGenCore facility. The DNA plasmids were propagated and purified using plasmid isolation kit (Qiagen, Valencia, CA, USA).

### Cell culture and DNA transfection

Pheochromocytoma (PC12, ATCC #CRL-1721) cells were maintained in Dulbecco’s modified eagle medium supplemented with 15 % horse serum, 2.5 % bovine calf serum, 2 mM glutamine and antibiotics (100 U/ml penicillin and 100 μg/mL streptomycin). Both cells were cultured at 37 °C in a 5% CO_2_ incubator. For hDAT (wild-type or mutant) transfection, cells were seeded into 24 well plates at a density of 1 × 10^5^ cells/cm^2^. After 24 h, cells were transfected with WT or mutant hDAT plasmids using Lipofectamine 2000 (Life Tech, Carlsbad, CA). Cells were used for the experiments after 24 h of transfection.

### [^3^H]DA uptake assay

Twenty four hours after transfection, [^3^H]DA uptake in PC12 cells transfected with WT-hDAT or mutant was performed according to the protocol reported previously^45^. To determine whether Tat inhibits DA uptake, kinetic analyses of [^3^H]DA uptake were conducted in WT hDAT and its mutants in the absence or presence of Tat. In brief, [^3^H]DA uptake was measured in Krebs-Ringer-HEPES (KRH) buffer (final concentration in mM: 125 NaCl, 5 KCl, 1.5 MgSO_4_, 1.25 CaCl_2_, 1.5 KH_2_PO_4_, 10 D-glucose, 25 HEPES, 0.1 EDTA, 0.1 pargyline, and 0.1 L-ascorbic acid; pH 7.4) containing one of 6 concentrations of unlabeled DA (final DA concentrations, 1.0 nM–5 μM) and a fixed concentration of [^3^H]DA (500,000 dpm/well, specific activity, 21.2 Ci/mmol; PerkinElmer Life and Analytical Sciences, Boston, MA). In parallel, nonspecific uptake of each concentration of [^3^H]DA (in the presence of 10 μM nomifensine, final concentration) was subtracted from total uptake to calculate DAT-mediated uptake. To determine the inhibitory effects of Tat on [^3^H]DA uptake, cells transfected with WT hDAT or mutant were preincubated with or without Tat_1–86_ (140 nM, final concentration) at room temperature for 20 min followed by addition of [^3^H]DA for an additional 8 min. The reaction was conducted at room temperature for 8 min and terminated by washing twice with ice cold uptake buffer. Cells were lysed in 500 μl of 1% SDS for an hour and radioactivity was measured using a liquid scintillation counter (model Tri-Carb 2900TR; PerkinElmer Life and Analytical Sciences, Waltham, MA). Kinetic data were analyzed using Prism 5.0 (GraphPad Software Inc., San Diego, CA).

## Additional Information

**How to cite this article**: Yuan, Y. *et al*. Role of Histidine 547 of Human Dopamine Transporter in Molecular Interaction with HIV-1 Tat and Dopamine Uptake. *Sci. Rep.* **6**, 27314; doi: 10.1038/srep27314 (2016).

## Supplementary Material

Supplementary Information

## Figures and Tables

**Table 1:** The calculated Y548−Y470 unbinding free energies and the detailed structural statistics of WT hDAT and its mutants in the outward-occluded state.

**Mutation**	**WT**	**H547A**	**H547P**	**Y470H**
Relative Y548-Y470 unbinding free energy (kcal/mol)^a^	4.65	5.78	1.70	0.00
Distance between the Cα atoms of Y470 and Y548 (Å)^b^,^c^	7.37 ± 0.40	7.26 ± 0.39	8.40 ± 0.50	9.57 ± 0.35
Cγ(D476)−Cζ(R85) distance in the D476-R85 salt bridge (Å)^b^,^d^	4.35 ± 0.36	4.25 ± 0.25	8.31 ± 0.42	7.61 ± 0.62
RMSD of Y470 Cα (Å)^b^,^e^	0.58 ± 0.23	0.44 ± 0.18	0.77 ± 0.24	0.54 ± 0.19
RMSD of R85 Cα (Å)^b^,^e^	0.63 ± 0.19	0.52 ± 0.20	0.60 ± 0.21	0.53 ± 0.21
RMSD of D476 Cα (Å)^b^,^e^	0.65 ± 0.25	0.52 ± 0.23	1.27 ± 0.28	1.17 ± 0.28

^a^The unbinding free energy required to separate the distance between Cα atoms of Y548 and Y470 from the lowest-energy distance to 10.25 Å (the common reference state).

^b^The statistics was based on the last 5 ns of the MD simulation for each protein system.

^c^The equilibrated distances between the Cα atoms of Y470 and Y548 in the outward-open, outward-occluded, and inward-open states of WT-hDAT were 7.37 ± 0.36 Å, 7.23 ± 0.35 Å, and 7.29 ± 0.37 Å, respectively. These data were based on the 50 ns MD trajectory for each system.

^d^The distances between the Cγ atom of D476 and Cζ atom of R85. The equilibrated distance in the D76-R85 salt bridge in the outward-open, outward-occluded, and inward-open states were 7.76 ± 0.57 Å, 4.30 ± 0.31 Å, and 4.05 ± 0.14 Å, respectively. These data were based on the 50 ns MD trajectory.

^e^The reference structure used in the RMSD calculations was the energy-minimized structure of WT hDAT in the outward-occluded state.

**Table 2:** Kinetic parameters (V_max_ and *K*_m_) for [^3^H]dopamine uptake by WT hDAT and its mutants.

	WT hDAT	H547A-hDAT	H547P-hDAT	Y470H-hDAT
V_max_ (pmol/min/10^5^ cells)	12.4 ± 1.51	36.7 ± 4.12^*^	0.2 ± 0.09^*^	2.2 ± 0.56^*^
*K*_m_ (μM)	1.3 ± 0.46	3.6 ± 1.47	2.6 ± 1.58	1.5 ± 0.08

Data are presented as mean ± S.E.M. values from five to seven independent experiments performed in duplicates. *p < 0.05 compared with WT hDAT (unpaired Student’s *t* test).
