# Supplementary material for: Role of Histidine 547 of Human Dopamine Transporter in Molecular Interaction with HIV-1 Tat and Dopamine Uptake
Source: Sci Rep. 2016 Jun 2;6:27314. doi: 10.1038/srep27314 (PMC4890318; doi:10.1038/srep27314)
Supplement: Supplementary Information [file srep27314-s1.pdf]

Supplementary Information

Role of Histidine 547 of Human Dopamine Transporter in Molecular Interaction with HIV-1 Tat and Dopamine Uptake

Yaxia Yuan,[a] Pamela M. Quizon,[b] Wei-Lun Sun,[b] Jianzhuang Yao,[a] Jun Zhu,[b] and Chang-Guo Zhan[a,]*

[a]*Molecular Modeling and Biopharmaceutical Center and Department of Pharmaceutical Sciences, College of Pharmacy, University of Kentucky, 789 South Limestone Street, Lexington, KY 40536 and* [b]*Department of Drug Discovery and Biomedical Sciences, South Carolina College of Pharmacy, University of South Carolina, 715 Sumter Street, Columbia, SC 29208*

Running title: Roles of Histidine 547 of Human Dopamine Transporter

Correspondence to:
Chang-Guo Zhan, Ph.D.
Director, Molecular Modeling and Biopharmaceutical Center (MMBC)
Director, Chemoinformatics and Drug Design Core of CPRI
Endowed College of Pharmacy Professor in Pharmaceutical Sciences
Professor, Department of Pharmaceutical Sciences
College of Pharmacy
University of Kentucky
789 South Limestone Street
Lexington, KY 40536
Phone: 859-323-3943
FAX: 859-257-7585
E-mail: zhan@uky.edu

* Corresponding author. E-mail: zhan@uky.edu

Additional Tables

Table S1. Sequence alignment of the YYY motif in important members of human NSS family. Alignment 1 indicates the sequence alignment from residue #467 to #473 of hDAT. Alignment 2 refers to the sequence alignment from residue #545 to #554 of hDAT. Conserved residues Y470, Y548, and Y551 in the alignment are colored in red, green, and blue, respectively.

Alignment 1	Alignment 2	Protein Name
GGIYVFT	PPHYG-AYIFP	DAT(Sodium-dependent dopamine transporter)
GGIYVLT	PLTYD-DYIFP	NET(Sodium-dependent noradrenaline transporter)
GGAYVVK	QLRLF-QYNYP	SERT(Sodium-dependent serotonin transporter)
AGIYWLL	PITYN-HYQYP	GlyT1(Sodium- and chloride-dependent glycine transporter 1)
GGIYMFQ	PMTYG-SYRYP	GlyT2(Sodium- and chloride-dependent glycine transporter 2)
GGIYVFK	PLTMG-NYVFP	GABA1(Sodium- and chloride-dependent GABA transporter 1)
GGMYVFQ	PLTYNKKYTYP	GABA2(Sodium- and chloride-dependent GABA transporter 2)
GGMYIFQ	PLKYNNIYTYP	GABA3(Sodium- and chloride-dependent GABA transporter 3)
GGMYWLV	PSEYG-SYRFP	PutP(Sodium-dependent proline transporter)
GGMYVFQ	PLVYNNTYVYP	CRT1(Sodium- and chloride-dependent creatine transporter 1)
GGMYVFQ	PLTYNKTYVYP	TauT(Sodium- and chloride-dependent taurine transporter)
GGMYIFQ	PLKYNNVYVYP	BetP(Sodium- and chloride-dependent betaine transporter)

Table S2. Sequence alignment of the YYY motif in dopamine transporter from different representative species. Alignment 1 indicates the sequence alignment from residue #467 to #473 of hDAT. Alignment 2 refers the sequence alignment from residue #545 to #554 of hDAT. Conserved residues Y470, Y548, and Y551 in the alignment are colored in red, green, and blue, respectively.

Alignment 1	Alignment 2	Species
GGIYVFT	PPHYGAYIFP	Homo sapiens (Human)
GGIYVFT	PPHYGAYIFP	Macaca fascicularis (Crab-eating macaque)
GGIYVFT	PPHYGAYIFP	Rattus norvegicus (Rat)
GGIYVFT	PPHYGAYVFP	Bos taurus (Bovine)
GGIYVFT	PPHYGAYIFP	Canis familiaris (Dog)
GGIYVFT	PPHYGDYVFP	Myotis lucifugus (Little brown bat)
GGIYVFT	SPKYGNYTFP	Xenopus tropicalis (Western clawed frog)
GGIYVFS	PPNYGSYIFP	Latimeria chalumnae (West Indian ocean coelacanth)
GGFYFFH	PLTYADYVYP	Drosophila melanogaster (Fruit fly)
GGFYFFQ	PLQYENYIYP	Bombyx mori (Silk moth)
GGFYFFH	PLTYEDYVYP	Apis mellifera (Honeybee)
GGFYFFH	PLSYEGYVYP	Lasius niger (Black garden ant)
GGILIME	ALTYQDYTYP	Caenorhabditis elegans
